# Endoscopic Drainage of Giant Pancreatic Pseudocysts Using Both Lumen-Apposing Metal Stent and Plastic Stent: A Report of Two Cases and Review of the Current Literature

**DOI:** 10.1155/2021/6610610

**Published:** 2021-04-02

**Authors:** Hussam I. A. Alzeerelhouseini, Muawiyah Elqadi, Mohammad N. Elqadi, Sadi A. Abukhalaf, Hazem A. Ashhab

**Affiliations:** ^1^Al-Quds University, Faculty of Medicine, Jerusalem, State of Palestine; ^2^Al-Ahli Hospital, Hebron, State of Palestine

## Abstract

**Introduction:**

A pancreatic pseudocyst (PP) with major diameter equal to 10 cm or more is called a giant pseudocyst. The ideal management for giant PPs is controversial. Endoscopic drainage is an alternative nonsurgical approach for PP management. Only a few cases of giant PPs were reported to be managed by endoscopic drainage. *Case Presentation*. We reported two cases of giant PPs following an episode of acute pancreatitis. Both were resolved following endoscopic cystogastrostomy using metallic and double-pigtail stents with excellent outcomes. There was no history of recurrence or complications on follow-up. In addition, we extensively reviewed all available literature studies of giant pancreatic pseudocyst presentation, management, and complications. We summarized all reported cases and presented them in a comprehensive table.

**Conclusion:**

The endoscopic cystogastrostomy approach is cost saving, can avoid surgical complications, and offers an early hospital discharge.

## 1. Introduction

Pancreatic pseudocyst (PP) is a peripancreatic fluid collection (PFC) and is a well-known complication of pancreatic diseases. PP usually occurs four weeks after acute pancreatitis and is a rich amylase fluid collection surrounded by a well-defined wall lacking solid material [1]. Worldwide, PP cases are increasing lately due to an increase in acute pancreatitis hospitalization over time [2] and advanced radiological modalities [3].

The management decision of PPs relies on clinical and imaging evaluation. A majority of PPs are asymptomatic with observation till spontaneous regression is required. However, symptomatic, persistent, large, or complicated pseudocysts require internal drainage [4, 5]. A pseudocyst with a major diameter equal to 10 cm or more is called a giant pseudocyst [6].

This intervention can be performed surgically or using less-invasive percutaneous or endoscopic approaches [7]. Endoscopic drainage of PPs is an alternative nonsurgical approach. Since the first reports by Sahel et al. [8] and Cremer et al. [9], endoscopic drainage of PPs has become an established procedure; it entails the creation of a fistulous tract between the PP and the gastric lumen (cystogastrostomy) or duodenal lumen (cystoduodenostomy) [8, 9].

Herein, we describe two cases in which giant pancreatic pseudocysts were resolved following endoscopic cystogastrostomy. Only a few cases of giant pancreatic pseudocysts were found in the literature review.

## 2. Case Presentation

### 2.1. Case 1

**Figure 1:**
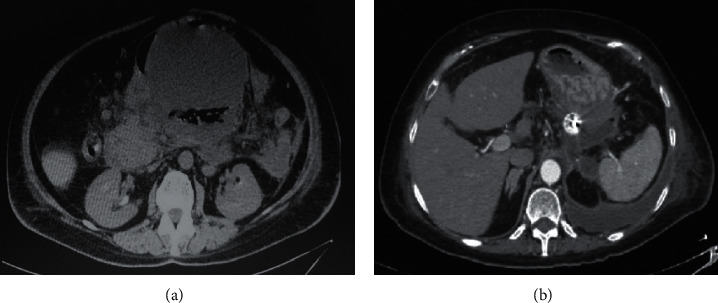
Abdominal CT scan showing (a) a huge pancreatic pseudocyst with air and fluid content and (b) resolution of most of the pseudocysts after AXIOS drainage.

A 63-year-old female was admitted to our hospital complaining of abdominal pain and vomiting. She was diagnosed as a case of acute idiopathic pancreatitis 4 weeks before her admission which was treated conservatively. On general examination, she was not icteric or feverish. Abdominal examination revealed a palpable, firm, tender epigastric mass and otherwise unremarkable. Laboratory investigations revealed markedly elevated serum amylase with normal lipase. White blood cell (WBC) count and liver enzymes were within normal limits. Chest X-ray (CXR) showed left-side moderate pleural effusion. An abdominal CT scan showed a huge pancreatic pseudocyst measuring 15 cm *∗* 15 cm *∗* 12 cm, occupying the body and tail of the pancreas (Figure 1(a)).

**Figure 2:**
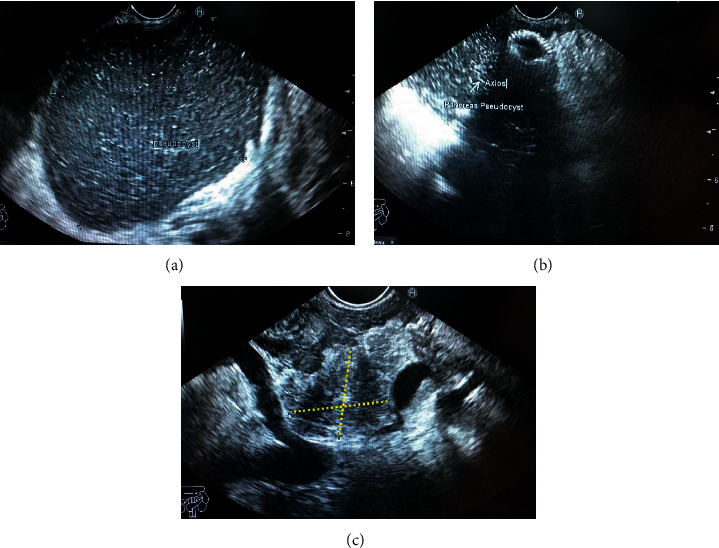
EUS image showing (a) a pancreatic pseudocyst before drainage and (b, c) the stent and pseudocyst after drainage (yellow dashed line).

**Figure 3:**
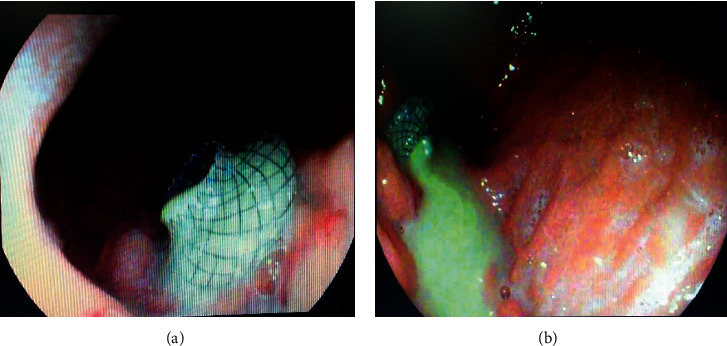
Endoscopic view showing AXIOS drainage of the pseudocyst.

Endoscopic ultrasound- (EUS-) guided drainage was performed as a therapeutic procedure using a Pentax linear echoendoscope. On the EUS, there was a huge fluid collection with air and debris consistent with the infected pancreatic pseudocyst (Figure 2(a)). A 10 mm length, 15 mm diameter Hot AXIOS stent was placed with EUS guidance with immediate drainage of >2000 cc of fluid and debris (Figures 2(b) and 2(c) and Figure 3). Then, a 10 French gauge, 1 cm long double-pigtail stent was placed under fluoroscopy guidance. The patient tolerated the procedure well, and there were no complications. One day after the procedure, a CT scan showed excellent results with regression of the pseudocyst. The patient was discharged home on antibiotics (Figure 1(b)).

**Figure 4:**
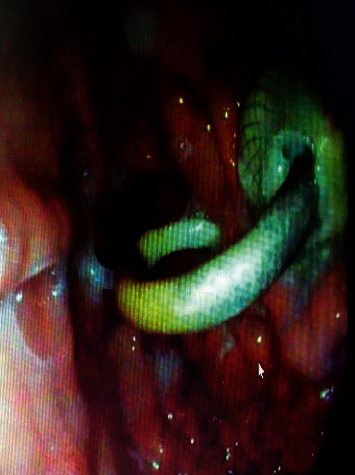
Hot AXIOS and double-pigtail stents at the time of removal.

On 6 weeks of follow-up, the patient reported disappearance of symptoms. EUS was repeated, and both AXIOS and plastic stents were removed (Figure 4); the patient made an uneventful recovery and was discharged home on antibiotics.

### 2.2. Case 2

A 37-year-old female was admitted to our hospital complaining of epigastric pain radiating to the back, vomiting, and early satiety. She had a history of acute biliary pancreatitis 4 weeks prior to her admission which responded well to medical management. On examination, there was marked epigastric distention and tenderness with decreased air entry at the base of the left lung. The examination of the other systems was normal. Laboratory tests revealed high serum lipase with normal serum amylase. Complete blood count (CBC), liver enzymes, and serum electrolytes were within normal limits.

**Figure 5:**
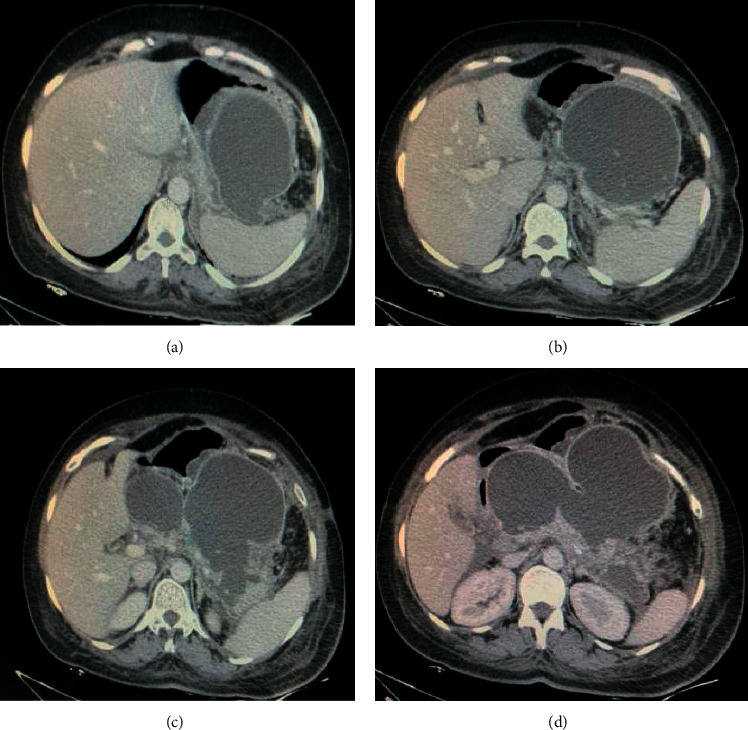
Contrast-enhanced CT scan of the abdomen—huge multiseptated pseudocyst occupying the body and tail of the pancreas.

A CXR revealed mild pleural effusion on the left side. An abdominal CT scan with contrast showed a giant multiseptated cyst measuring about 20 *∗* 15 *∗* 8 cm in the body and tail of the pancreas consistent with the pancreatic pseudocyst (Figure 5). The head of the pancreas was enlarged and surrounded by significant fat stranding. There was a moderate amount of free fluid noted at the Morison pouch and pelvic cavity. The liver, the spleen, and both kidneys were normal in size and density.

**Figure 6:**
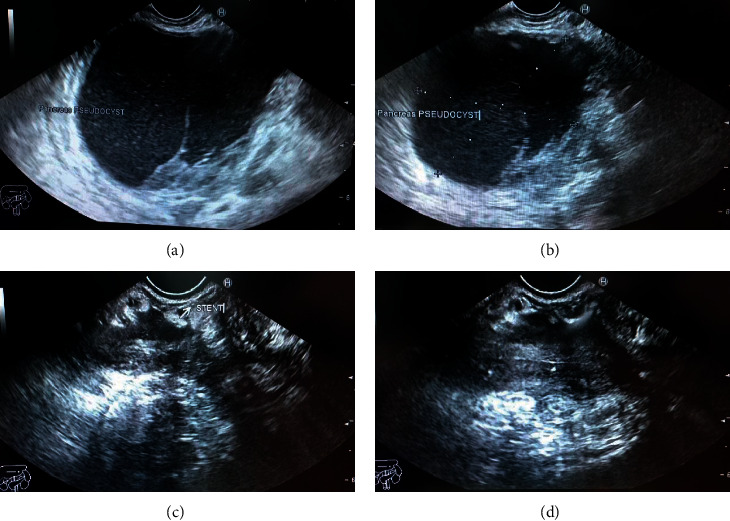
EUS image showing (a, b) a pancreatic pseudocyst before drainage and (c, d) the stent and pseudocyst after drainage.

**Figure 7:**
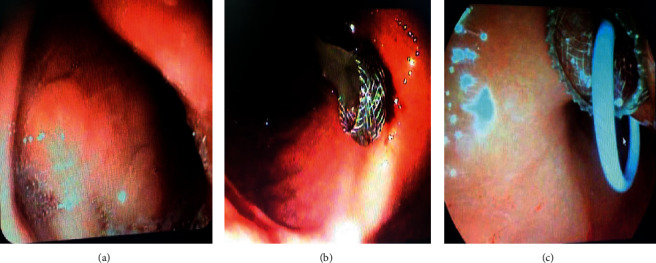
Endoscopic view during pseudocyst drainage showing (a) the pseudocyst bulging the gastric mucosa, (b) drainage from the pseudocyst, and (c) AXIOS and double-pigtail stents.

**Figure 8:**
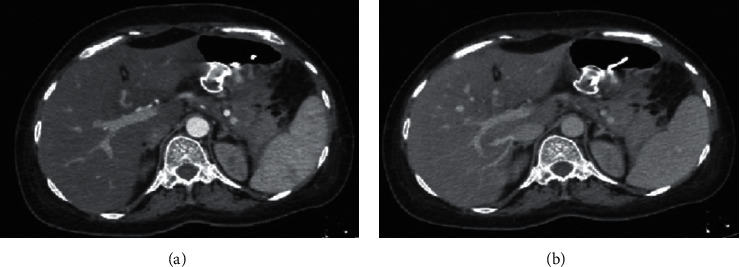
Contrast-enhanced CT scan reveals effective drainage of the pseudocyst.

Endoscopic ultrasound- (EUS-) guided drainage was performed to relieve this giant symptomatic pseudocyst. Periprocedural period was covered with an antibiotic. EUS showed a huge extrinsic bulge at the body and antrum part of the stomach. The Hot AXIOS stent with 10 mm *∗* 15 mm diameter was placed with excellent drainage of >1500 cc of fluid, and then a 10 French gauge, 3 cm long double-pigtail stent was placed under fluoroscopy guidance (Figures 6 and 7). The follow-up CT scan demonstrated almost total regression of the pseudocyst (Figure 8). The patient made an uneventful recovery and was discharged home on the same day on antibiotics.

On 6 weeks of follow-up, the patient was doing well. Repeated EUS showed full resolution of the pseudocyst, and both stents were removed without complications.

## 3. Discussion

Pancreatic fluid collections (PFCs), according to the revision of the Atlanta classification of acute pancreatitis, are described in four subtypes: acute peripancreatic fluid collection, acute necrotic collection, pancreatic pseudocyst (PPs), and walled-off necrosis (WON) [1]. Pancreatic pseudocysts are collections of high concentrations of digestive enzymes in the retroperitoneum or the peripancreatic tissue planes that are lined by fibrous tissues and may contain necrotic debris or blood but lack a true epithelial lining [2]. Pancreatic pseudocysts usually appear 4 weeks after an episode of chronic pancreatitis, acute pancreatitis, or disruption in the pancreatic duct such as blunt, penetrating trauma, or injury during surgeries [3, 10, 11] (Table 1). PPs' incidence is relatively low [29], and they are more in chronic pancreatitis compared to acute pancreatitis [30]. PPs present usually singular in the head of the pancreas though multiple PPs may present as well [11].

Most pseudocysts are small, asymptomatic, and diagnosed incidentally. However, pseudocysts can produce a wide range of clinical manifestations that can be usually ascribed to the local mass effect of the pseudocyst or the associated inflammatory response. Depending on location and size, clinical symptoms may include abdominal pain, early satiety, weight loss, persistent fevers, infection of the pseudocyst, duodenal or biliary obstruction, vascular occlusion, free rupture of the pseudocyst into the peritoneal cavity, fistula formation, or digestion of an adjacent vessel resulting in pseudoaneurysms [10]. Our study on giant pseudocysts reported abdominal pain in 91% of patients, abdominal distention in 32%, abdominal mass in 22.7%, anorexia, early satiety in 18%, weight loss in 13.6%, and vomiting and fever in 9% (Table 2).

The diagnosis of pseudocysts is increased as radiologic methods have developed [3]. The diagnosis is usually made by CT scan, magnetic resonance imaging (MRI), or transabdominal ultrasound (US). PPs appear as intra- or peripancreatic encapsulated fluid collections [11]. Findings on the CT scan of the pancreatic pseudocyst according to the revision of the Atlanta classification of acute pancreatitis include a well-circumscribed fluid collection that is usually round or oval, the fluid collection is typically extrapancreatic, homogenous fluid density, no nonliquid components within the fluid, and a well-defined wall that completely encapsulates the fluid collection [1]. If the diagnosis is in doubt, it is crucial to differentiate between the pseudocyst and a cystic pancreatic neoplasm; sampling of the cyst fluid may be necessary [31].

To date, there is no consensus on the ideal management of pancreatic pseudocysts. Although there are no crystal clear guidelines, pancreatic pseudocysts were traditionally managed with open surgical approaches including cyst-gastrostomy, cyst-duodenostomy, Roux-en-Y cyst-jejunostomy, and external drainage [10]. Recently, due to the advancement in the endoscopic era, endoscopic drainage of pancreatic pseudocysts has been performed with variable success rates though comparable studies to the surgical approaches have not been launched, and thus, management certainty cannot be achieved [10]. A 10-patient study has demonstrated that there are no significant differences between endoscopic drainage of pancreatic pseudocysts and surgical approaches in terms of treatment success, procedural complications, or reintervention. The significant differences observed were in-hospital length of stay and the mean cost savings [32]. Another study had reported a higher complication rate in the endoscopic drainage of the pancreatic pseudocyst group [33].

One of the largest related studies had demonstrated that endoscopic drainage may be performed in acute or chronic pancreatic pseudocysts and pancreatic necrosis though with different rates of successful drainage, complications, and recurrence. The study reported that chronic pancreatic pseudocysts were found to be resolved higher than acute pancreatic pseudocysts or pancreatic necrosis groups and as a marker for a successful drainage. The authors found that pancreatic necrosis was a marker for lower success rates and higher rates of complications and recurrence [5].

Endoscopic drainage of pancreatic pseudocysts has been performed using both transpapillary and transenteric approaches. To allow complete resolution, the transpapillary approach requires that the pseudocyst communicates with the main pancreatic duct and has few septations to permit complete drainage [10]. The transenteric approach can be achieved using a gastric or duodenal wall after confirming the adherence between the selected wall and the cyst using either endoluminal bulge or endoscopic ultrasound [10]. The cyst adherence is strictly associated with cyst size; larger and huge cysts may be accessed more easily though may need a longer time to complete resolution.

The pancreatic pseudocyst that measured >10 cm in major diameter was defined as a giant pancreatic pseudocyst [6]. There are several reported giant pancreatic pseudocyst cases in the literature (Table 1), with only a few cases reporting management [3, 12, 13, 15, 18, 34]. Surgical cystogastrostomy was the most common used approach; Wang and Misra [13] and Report [15] described the surgical midline incision approach to drain giant pancreatic pseudocysts measured more than 25 cm in the major diameter; it drained 3 L and 4.5 L, respectively, and both patients recovered uneventfully. The other used less invasive methods for huge pancreatic pseudocysts included percutaneous or endoscopic cystogastrostomy and percutaneous CT-guided cystogastrostomy. Alhassan et al. [34] reported percutaneous CT-guided cystogastrostomy with good outcomes and no complications with one-year follow-up. Regarding endoscopic cystogastrostomy, it may not be appropriate for the giant pseudocyst as an initial approach in the absence of well-trained hands, and using other options may be safer [3].

Over the last decade, EUS-guided drainage has been conventionally performed for the peripancreatic fluid collection (PFC) with a plastic stent such as double-pigtail stent (DPS) and a fully covered self-expanding metal stent. However, more recently, a dedicated device, a lumen-apposing metal stent (LAMS), has been developed as an alternative for PFC [35]. LAMS has a larger-diameter lumen (10 or 15 or newer 20 mm) compared with DPSs, and this can allow more efficient drainage with less risk of stent occlusion and superimposed infection. Moreover, LAMSs are designed with a biflanged shape that allows for tissue apposition and minimizes the risk of stent migration. Also, it can provide the channel for endoscopic necrosectomy and the need for repeated endoscopies [36]. There is a reduced risk of leakage with the LAMS compared to plastic stents mainly due to dual flange anchors and the fully covered nature of the LAMS [37]. Risk of perforations is more common in the DPS than in the LAMS, which some providers attributed to the fact that dual DPSs are technically more difficult to place than LAMSs [38].

The clinical and technical success rates of LAMSs have been reported to be 93–100% and 89–100%, respectively [36], with complication rates around 10%–15% [39]. LAMSs have become the stent of choice for endoscopic drainage of PFCs by many gastroenterologists because of their easy deployment, less procedure time, and direct debridement access [36]. Several studies demonstrated that LAMSs have better efficacy and safety over plastic stents for PFC [40].

The main drawback of the LAMS is that it was found to be associated with a higher bleeding rate when compared to the DPS, including late bleeding at 3–5 weeks in the treatment of postpancreatitis pancreatic fluid collections [41]. EUS guidance with the use of color Doppler may reduce the risk of intraprocedural bleeding but would not affect delayed bleeding [42]. One hypothesis is that the LAMS remained in place even after the collapse of the collection without any movement, and this causes friction to the surrounding vasculature around the necrotic cavity promoting pseudoaneurysm formation and subsequent bleeding [41]. Another explanation is the easier access of low-pH fluid with gastric acid into the cyst cavity due to the wider lumen of the LAMS, thereby causing irritation of exposed intracavitary vessels and an increased tendency for bleeding [43]. This prompted gastroenterologists to change their practice of repeating imaging at 3 weeks to assess the cavity resolution instead of 6 weeks, followed by stent removal if the fluid collection is resolved [37].

Current literature review revealed that the LAMS with coaxially placed DPS for PP drainage has been reported to be associated with a lower risk of bleeding [39] and low rates of infection compared to the placement of the LAMS alone (0% vs. 17%) [44]. A possible explanation for these lower risks is mainly due to anchoring LAMS with coaxial plastic stent which can make the passage of solid particles and low gastric pH more difficult while continuing to allow for drainage of liquid secretions around the pigtail stent [37].

## 4. Conclusion

The ideal management for giant pancreatic pseudocysts is controversial. Only a few cases of giant pancreatic pseudocysts were reported to be managed by endoscopic drainage. Endoscopic drainage is an alternative nonsurgical approach for pancreatic pseudocysts' management that lately gained a wide range of acceptance due to its lower morbidity, mortality, and costs.

Most studies showed the superiority of the lumen-apposing metal stent on the double-pigtail stent in the management of peripancreatic fluid collection. The literature showed that the addition of a coaxial double-pigtail stent to the lumen-apposing metal stent was associated with a lower rate of adverse events in endoscopic drainage of peripancreatic fluid collection.

## Figures and Tables

**Table 1:** Characteristics of the most reported giant pancreatic pseudocysts (largest dimension of at least 10 cm) in the literature.

Study reference	Age	Sex	Presentation	Cause of the pseudocyst	Pleural effusion	Time after pancreatitis	WBC	Amylase	Lipase	CEA and CA19-9	Cyst size on CT or US in cm (largest dimension)	Diagnosis	Management	Complications	Second drainage needed	Discharge time	Type of stent used	Time of stent removal after EUS	Necrosectomy needed
[12]	33	F	Abdominal pain Abdominal distention Anorexia	Acute pancreatitis	—	—	Normal	Elevated	—	Normal	10.3 *∗* 9.6 *∗* 9.3	Abdominal computed tomography (CT)	Open retrogastric cystogastrostomy	No	—	Day 7	—	—	—
[13]	65	M	Abdominal pain Abdominal distention Anorexia Weight loss	Acute pancreatitis	—	—	Elevated	—	Elevated	Normal	25.7 *∗* 15.3 *∗* 10.9	Abdominal computed tomography (CT)	Open retrogastric cystogastrostomy	Yes (bleeding in day 4 POD)	—	Day 6	—	—	—
[14]	16	M	Abdominal pain Abdominal distention Dyspnea	—	Yes (bilateral)	—	—	Elevated	—	—	35 *∗* 15 *∗* 14	Diagnostic laparoscopy	Laparotomy (excision of the pseudocyst and Roux-en-Y pancreaticojejunostomy)	No	—		—	—	—
[15]	56	M	Abdominal pain Abdominal distension Abdominal mass	Acute pancreatitis	Yes (right)	6 weeks	—	—	—	—	25 *∗* 17	Abdominal computed tomography (CT)	Open cystogastrostomy	No	—	10 pod	—	—	—
[16]	63	F	Abdominal distention	Acute pancreatitis	—	6 weeks	—	—	—	—	12	Abdominal computed tomography (CT)	Open cystogastrostomy	No	—	8 pod	—	—	—
[17]	39	M	Abdominal pain	Acute pancreatitis	—	—	—	Elevated	Elevated	—	10 *∗* 8	Abdominal computed tomography (CT)	Laparoscopic cystogastrostomy	No	—	21 pod	—	—	—
[18]	37	F	Abdominal pain Abdominal mass	Acute pancreatitis	—	12 weeks	Normal	—	—	—	23 *∗* 15 *∗* 12	Abdominal computed tomography (CT)	Laparoscopic transgastric cystogastrostomy	Yes (pseudocyst infection)	—	9 pod	—	—	—
[19]	18	M	Abdominal mass	Abdominal trauma	—	—	—	—	—	—	22 *∗* 18	Abdominal computed tomography (CT)	Laparoscopic transgastric cystogastrostomy	No	—	12 pod	—	—	—
[20]	49	M	—	Acute pancreatitis	—	—	—	Elevated	—	Normal	10 *∗* 4	Abdominal computed tomography (CT)	Laparoscopic cystogastrostomy	No	—		—	—	—
[21]	48	M	Abdominal pain Abdominal mass Anorexia Early satiety Weight loss	Chronic pancreatitis	—	—	Normal	Elevated	Elevated	Normal	21	Abdominal computed tomography (CT)	CT-guided percutaneous drainage	No	—	—	—	—	—
[22]	61	M	—	Acute pancreatitis	—	—	Elevated	—	Elevated	—	13	Abdominal computed tomography (CT)	EUS-guided drainage	Yes (recurrence)	Yes		Metallic stent	8 weeks	Yes
[22]	65	M	Abdominal pain	Chronic pancreatitis	—	—	Elevated	—	—	—	10 *∗* 8.2 *∗* 4.6	Abdominal computed tomography (CT)	EUS-guided drainage	No	No	6 days	Metallic stent	6 weeks	No
[22]	61	F	Abdominal pain	Acute pancreatitis	—	—	Elevated	—	—	—	14	Abdominal computed tomography (CT)	EUS-guided drainage	Yes (recurrence)	Yes	10 days	1-metallic stent 2-plastic double-pigtail stent		No
[22]	53	M	Abdominal pain Vomiting	Acute pancreatitis	—	7 weeks	—	Elevated	Elevated	—	10 *∗* 12 *∗* 8.7	Abdominal computed tomography (CT)	EUS-guided drainage	No	No	6 days	Metallic stent	2 weeks	No
[23]	50	M	Abdominal pain Abdominal mass Vomiting	Acute pancreatitis	Yes (right)	20 weeks	Normal	Elevated	—	—	12.7 *∗* 10.8	Abdominal US Abdominal computed tomography (CT)	EUS-guided drainage	No	No	7 days	—	—	No
[24]	37	M	Abdominal pain	Acute pancreatitis	—	4 weeks	Normal	Elevated	—	Normal	10 *∗* 7	Abdominal US	EUS-guided drainage	No	No	Same day	Plastic double-pigtail stent	—	No
[24]	54	F	Abdominal pain Fever	Acute pancreatitis	—	—	Normal	Elevated	Elevated	Normal	12 *∗* 7 *∗* 8	Abdominal computed tomography (CT)	EUS-guided drainage	No	No	—	Plastic double pigtail stent	3 weeks	No
[25]	54	F	Abdominal pain	Acute pancreatitis	—	3 weeks	Normal	—	—	—	13 *∗* 14	Abdominal computed tomography (CT)	EUS-guided drainage	Yes (stent migration into pseudocyst)	No	Same day	Lumen-apposing metal stent	5 weeks	No
[26]	81	M	Abdominal pain	Acute pancreatitis	No	4 weeks	—	Elevated	—	—	17 *∗* 11	Abdominal computed tomography (CT)	EUS-guided drainage	Yes (recurrence)	Yes	—	Plastic double-pigtail stent	8 weeks	No
[27]	74	M	Abdominal pain Fever	Chronic pancreatitis	Yes (bilateral)	—	—	—	—	—	10	Abdominal computed tomography (CT)	EUS-guided drainage	No	No	Same day	Plastic double-pigtails stent	—	No
[3]	27	M	Abdominal pain Abdominal distention Weight loss Anorexia	Acute pancreatitis	Yes (right)	8 weeks	Normal	Elevated	—	—	30 *∗* 15 *∗* 14	Abdominal computed tomography (CT)	EUS-guided drainage	Yes (recurrence)	Yes (after 1 week)	Same day	1-lumen-apposing metal stent 2-plastic double-pigtail stent	3 weeks	No
[28]	69	M	Abdominal pain Abdominal mass	Chronic pancreatitis	—	1 year	Normal	Normal	Elevated	—	18	Abdominal computed tomography (CT)	EUS-guided drainage	Yes (pseudocyst infection, stent migration, and recurrence)	Yes	Same day	Plastic double-pigtail stent	8 weeks	Yes
This study	63	F	Abdominal pain Abdominal mass Vomiting	Acute pancreatitis	Yes (left)	3 weeks	Normal	Elevated	Normal	Normal	15 *∗* 15 *∗* 12	Abdominal computed tomography (CT)	EUS-guided drainage	No	No	Same day	1-lumen-apposing metal stent 2-plastic double-pigtail stent	9 weeks	Yes
This study	37	F	Abdominal pain Abdominal distension Vomiting Early satiety	Acute pancreatitis	Yes (left)	4 weeks	Normal	Normal	Elevated	Normal	20 *∗* 15 *∗* 8	Abdominal computed tomography (CT)	EUS-guided drainage	No	No	Same day	1-lumen-apposing metal stent 2-plastic double-pigtail stent	6 weeks	No

**Table 2:** Characteristics of the 24 most reported giant pancreatic pseudocysts.

Variable	Value
Age	Mean: 50.4 years, range: 16–81
Male : female ratio	1 : 0.5
Cause	
Acute pancreatitis	18/23 (78%)
Chronic pancreatitis	4/23 (17.4%)
Trauma	1/23 (4.3%)
Clinical presentation	
Time of pseudocyst presentation after acute pancreatitis	Mean: 7 weeks, range: 3–20 weeks
Abdominal pain	20/22 (91%)
Abdominal distention	7/22 (32%)
Abdominal mass	5/22 (22.7%)
Anorexia	4/22 (18%)
Early satiety	4/22 (18%)
Weight loss	3/22 (13.6%)
Vomiting	2/22 (9%)
Fever	2/22 (9%)
Laboratory investigations	
Elevated WBC	4/15 (26%)
Elevated amylase or lipase	16/16 (100%)
Normal CEA and CA19-9	8/8 (100%)
Diagnosis	
Abdominal CT scan	22/24 (92%)
Abdominal US	1/24 (4%)
Diagnostic laparoscopy	1/24 (4%)
Cyst size on CT or US (largest dimension)	Mean: 16.7 cm (10–35 cm)
Management	
*(1) EUS-guided drainage (14 cases)*	
Time for stent removal after EUS	Mean: 5.8 weeks, range: 2–9 weeks
Types of stents used	
Metallic	4/13
Plastic (double pigtail)	5/13
Both	4/13
Complications	
Recurrence of the pseudocyst	5/14 need second drainage (35%), and only 1 case needs third drainage (7%)
Pseudocyst infection	1/14 (7%)
Stent migration	2/14 (14%)
Necrosectomy needed	3/14 (21%)
Time of discharge	Mean: 2.6 days
*(2) Open surgery or laparoscopic (9 cases)*	
Time of discharge	Mean: 10 days

CEA: carcinoembryonic antigen; EUS: endoscopic ultrasound.

## Data Availability

The data used to support the findings of this study are available from the corresponding author upon reasonable request.
